# Artificial intelligence-based ^68^Ga-DOTATOC PET denoising for optimizing ^68^Ge/^68^Ga generator use throughout its lifetime

**DOI:** 10.3389/fmed.2023.1137514

**Published:** 2023-03-13

**Authors:** Elske Quak, Kathleen Weyts, Cyril Jaudet, Anaïs Prigent, Gauthier Foucras, Charline Lasnon

**Affiliations:** ^1^Nuclear Medicine Department, Comprehensive Cancer Centre François Baclesse, UNICANCER, Caen, France; ^2^Radiophysics Department, Comprehensive Cancer Centre François Baclesse, UNICANCER, Caen, France; ^3^Radiopharmacy Department, Comprehensive Cancer Centre François Baclesse, UNICANCER, Caen, France; ^4^UNICAEN, INSERM 1086 ANTICIPE, Normandy University, Caen, France

**Keywords:** PET, gallium-68, artificial intelligence, denoising, deep learning

## Abstract

**Introduction:**

The yield per elution of a ^68^Ge/^68^Ga generator decreases during its lifespan. This affects the number of patients injected per elution or the injected dose per patient, thereby negatively affecting the cost of examinations and the quality of PET images due to increased image noise. We aimed to investigate whether AI-based PET denoising can offset this decrease in image quality parameters.

**Methods:**

All patients addressed to our PET unit for a ^68^Ga-DOTATOC PET/CT from April 2020 to February 2021 were enrolled. Forty-four patients underwent their PET scans according to Protocol_FixedDose (150 MBq) and 32 according to Protocol_WeightDose (1.5 MBq/kg). Protocol_WeightDose examinations were processed using the Subtle PET software (Protocol_WeightDose^AI^). Liver and vascular SUV mean were recorded as well as SUVmax, SUVmean and metabolic tumour volume (MTV) of the most intense tumoural lesion and its background SUVmean. Liver and vascular coefficients of variation (CV), tumour-to-background and tumour-to-liver ratios were calculated.

**Results:**

The mean injected dose of 2.1 (0.4) MBq/kg per patient was significantly higher in the Protocol_FixedDose group as compared to 1.5 (0.1) MBq/kg for the Protocol_WeightDose group. Protocol_WeightDose led to noisier images than Protocol_FixedDose with higher CVs for liver (15.57% ± 4.32 vs. 13.04% ± 3.51, *p* = 0.018) and blood-pool (28.67% ± 8.65 vs. 22.25% ± 10.37, *p* = 0.0003). Protocol_WeightDose^AI^ led to less noisy images than Protocol_WeightDose with lower liver CVs (11.42% ± 3.05 vs. 15.57% ± 4.32, *p* < 0.0001) and vascular CVs (16.62% ± 6.40 vs. 28.67% ± 8.65, *p* < 0.0001). Tumour-to-background and tumour-to-liver ratios were lower for protocol_WeightDose^AI^: 6.78 ± 3.49 vs. 7.57 ± 4.73 (*p* = 0.01) and 5.96 ± 5.43 vs. 6.77 ± 6.19 (*p* < 0.0001), respectively. MTVs were higher after denoising whereas tumour SUVmax were lower: the mean% differences in MTV and SUVmax were + 11.14% (95% CI = 4.84–17.43) and −3.92% (95% CI = −6.25 to −1.59).

**Conclusion:**

The degradation of PET image quality due to a reduction in injected dose at the end of the ^68^Ge/^68^Ga generator lifespan can be effectively counterbalanced by using AI-based PET denoising.

## Background

The half-life of the ^68^Ga isotope is short (68 min) requiring on-site synthesis of ^68^Ga-labeled tracers. The advent of commercially available ^68^Ge/^68^Ga generators and labeling kits has facilitated the synthesis of ^68^Ga-labeled PET tracers in the hospital’s radiopharmacy and contributed to its increased use. Frequently used ^68^Ga-labeled PET tracers target somatostatin receptors in neuroendocrine tumours (NETs) (1) and prostate-specific membrane antigen (PSMA) in prostate cancer (2). The clinical benefits of ^68^Ga-labeled PET tracers for imaging and diagnosis of NETs include improved sensitivity and specificity compared to other imaging modalities, as well as the ability to detect small and functional tumours. It is recommended as the first choice for PET/CT imaging of most NETs by international guidelines (3–6). Since the half-life of the parent ^68^Ge isotope is 271 days, the generator lifespan is about 1 year. At the start of the lifespan, one generator elution allows the labeling of approximately four doses based on an injected dose of 3 MBq/kg. However, as the ^68^Ge parent of the generator decays over time, the number of doses of tracer obtained per elution decreases. This means that during the lifespan of the generator, the number of examinations per elution and/or the activity injected in the patient in MBq/kg decreases, thereby negatively affecting the cost of the procedure or the quality of PET images due to increased image noise. Moreover, due to the short half-life of ^68^Ga, the increase in image noise can hardly be counterbalanced by an increase in PET acquisition time, particularly if several patients injected with the same elution need to be scanned.

To optimize the use of the ^68^Ge/^68^Ga generator while maintaining PET image quality, innovative approaches based on artificial intelligence (AI) are opening up new perspectives. By using AI, the acquisition time per exam and/or the injected activity can be reduced without compromising image quality. Notably, several AI-based post-reconstruction PET/CT image enhancements have been recently developed (7). A post-reconstruction PET denoising software (SubtlePET™, Subtle Medical©, Stanford, USA provided by Incepto©, France) that was recently developed by using a deep convolutional neural network on a library of millions of paired images (native and low-dose images) to learn and tune the optimal parameters to compute an estimate of the native image. Currently, only a few clinical publications have evaluated its use in oncology, all of them dealing with ^18^F-FDG PET images (8–12). At present, SubtlePET™ is FDA (Food and Drug Administration)-approved for use with 18F-FDG and 18F-Amyloid tracers and is now CE (European Conformity)-marked for use with 18F-FDG, 18F-Amyloid, 18F-Fluciclovine, 18F-DOPA, 18F-Choline, 18F-DCFPyL, Ga-68 Dotatate, and Ga-68 PSMA PET images (13). However, no clinical study has demonstrated the value of this software to enhance the quality of low-dose ^68^Ga PET images, even though nuclear medicine departments are concerned about this issue. Various other deep learning-based methods have been evaluated for low-dose imaging and resolution enhancement, but none of them are currently validated for clinical use (14). Denoising techniques for ^68^Ga-labeled radiotracers in PET imaging have been explored using both reconstruction-based methods and deep-learning techniques. It has been shown that both strategies can significantly improve the image quality by decreasing the noise level in low-dose ^68^Ga PET scans (15).

Therefore, the aim of this prospective study was to explore the performance of this software to enhance the quality of ^68^Ga-DOTATOC PET images, and to compare it to a standard Gaussian post-filtering approach. We hypothesized that to optimize the use of a ^68^Ge/^68^Ga generator throughout its lifetime, AI-based PET denoising might be a solution to maintain correct image quality.

## Materials and methods

### Population

All patients were informed about the use of their clinical and PET data for research purposes. Patients had the right to refuse the transmission of data covered by medical confidentiality used and processed in the context of this research. The procedure was declared to the National Institute for Health Data with the registration no. F20210720123322. Patients over 18 years old addressed to our PET unit for a ^68^Ga-DOTATOC PET from April 2020 to February 2021 were enrolled. Sex, age and body mass index (BMI) were extracted from electronic patient records.

### Positron emission tomography acquisition and reconstruction

All patients underwent their examinations on a VEREOS PET/CT system (Phillips). All PET emission acquisitions were performed 60 min after injection, from the skull to mid-thighs with 1 min 30 per bed position. Images were reconstructed with four iterations four subsets with point spread function (PSF) and 2-mm voxel size. All images were acquired and reconstructed according to the European guidelines (16). In the event of treatment with somatostatin analogs, the treatment was stopped at least 21 days before the PET scan.

Between April and November 2020, corresponding to the first months of the generator’s lifespan, patients were injected intravenously with a fixed dose of 150 MBq of ^68^Ga-DOTATOC. This protocol is subsequently referred to as *protocol_FixedDose*.

Between December 2020 and February 2021, i.e., the last months of the generator’s lifespan, patients were injected intravenously with 1.5 MBq/kg of ^68^Ga-DOTATOC. This protocol is subsequently referred to as *protocol_WeightDose*. These PET examinations were then processed using Subtle PET™software and was subsequently referred to as *protocol_WeightDose^AI^*.

In addition, NEMA-NU2 image quality phantom acquisitions were performed and analyzed to find a specific Gaussian post-filter (GPF). This GPF will allow the *protocol_WeightDose* to recover a noise in the image equivalent to the former *protocol_FixedDose* (17). Measurements were made with a sphere-to-background ratio set at six and two background ^68^Ga solution concentrations: 2.1 MBq/mL and 1.5 MBq/mL, corresponding to the average injected activities for *protocol_FixedDose* and *protocol_WeightDose*, respectively. CVs were measured in a VOI larger than 100 ml for both acquisitions. The width of the fitted GPF was optimized by dichotomy. This GPF was then applied to all *protocol_WeightDose* acquisitions and the resulting images referred to as *protocol_WeightDose^Gaussian^*.

### Clinical PET data extraction

Positron emission tomography scans were equally and randomly assigned to two senior nuclear physicians. PET images were reviewed on MIM (MIM Software, Cleveland, OH, USA, version 5.6.5).

The following features were recorded separately for each PET acquisition:

• Liver SUV_mean_ (mean standard uptake value) and standard deviation (SD) from a 3 cm diameter spherical volume of interest (VOI) placed on the right liver lobe.
• Vascular SUV_mean_ and SD from a 2 cm diameter spherical-VOI placed on the descending aorta.
• Muscular SUV_mean_ and SD from a 2 cm spherical-VOI placed on the left erector spinae muscle at the height of the adrenals.
• Tumour SUV_max_, SUV_mean_ and metabolic tumour volume (MTV) from a 40% isocontour VOI placed on the most intense lesion, as well as its location.
• The tumour background SUV_mean_ from a doughnut-shaped VOI surrounding the most intense lesion VOI.

Physiological noises were evaluated by means of coefficients of variations (CV) calculated as follows: $\frac{S\,D}{S\,U\,V_{\text{mean}}}\times 100\left(\%\right)$. Lesion-to-background ratios were computed as follows: $\frac{t\,u\,m\,o\,r\,S\,U\,V_{\text{mean}}}{b\,a\,c\,k\,g\,r\,o\,u\,n\,d\,S\,U\,V_{\text{mean}}}$.

### Statistical analysis

Data was presented as mean (SD) unless otherwise specified.

Unmatched data were compared using Mann–Whitney and Kruskal–Wallis tests for quantitative data as appropriate. Wilcoxon and Friedman tests, and Bland–Altman analyses were used to compare paired quantitative data as appropriate.

Statistical analysis and figure design were performed using XLSTAT software (XLSTAT 2019: Data Analysis and Statistical Solution for Microsoft Excel, Addinsoft). *P*-values < 0.05 were considered statistically significant.

## Results

### Population characteristics

Sixty-seven patients were included. Forty-four patients underwent their PET scans according to *protocol_FixedDose* and 32 according to *protocol_WeightDose*. Of note, nine patients underwent both protocols to monitor their disease over the inclusion period. Patients’ characteristics can be found in Table 1. Age, sex, BMI, PET indications and uptake delay were not different between *protocol_FixedDose* and *protocol_WeightDose* groups. The mean injected dose of 2.1 (0.4) MBq/kg per patient was significantly higher in the *protocol_FixedDose* group as compared to 1.5 (0.1) MBq/kg for the *protocol_WeightDose* group. Using the *protocol_FixedDose*, 93% of patients were injected with more than 1.5 MBq/kg, with an injected dose ranging from 1.4 MBq/kg in a severely obese patient (BMI = 41.2 kg/m^2^) to 3.0 MBq/kg injected in a normal weight patient (BMI = 19.1 kg/m^2^) (Supplementary Figure 1).

**TABLE 1:** Patients and PET examination characteristics.

Variables	*Protocol_FixedDose* (*n* = 44)	*Protocol_WeightDose* (*n* = 32)	*P*-value*
**Patient characteristics**			
**Sex, *n* (%)**			
• Female	18 (40.9)	18 (54.5)	0.246
• Male	26 (59.1)	14 (44.5)	
Age (yrs.), mean (SD)	65 (10)	63 (12)	0.521
BMI (kg/m^2^), mean (SD)	25.7 (4.6)	25.4 (7.4)	0.858
**PET indications, *n* (%)**			
• Staging	7 (15.9)	7 (21.9)	0.545
• Disease monitoring	21 (47.7)	19 (59.4)	
• Suspected recurrence	3 (6.8)	2 (6.2)	
• Before PRRT	3 (6.8)	1 (3.1)	
• Metabolic lesion characterization	10 (22.7)	3 (9.4)	
**PET examination characteristics**			
Injected dose per patient (MBq), mean (SD)	151.6 (13.0)	111.8 (27.3)	<0.0001
Injected dose per patient (MBq/kg), mean (SD)	2.1 (0.4)	1.5 (0.1)	<0.0001
Uptake delay (min), mean (SD)	59 (5)	58 (3)	0.288

*Non-parametric Mann–Whitney tests *p*-values, except for PET indications and sex for which Fisher exact tests were performed. BMI, body mass index; PRRT, peptide receptor radionuclide therapy.

### Comparison of *protocol_FixedDose* and *protocol_WeightDose*

**FIGURE 1:**
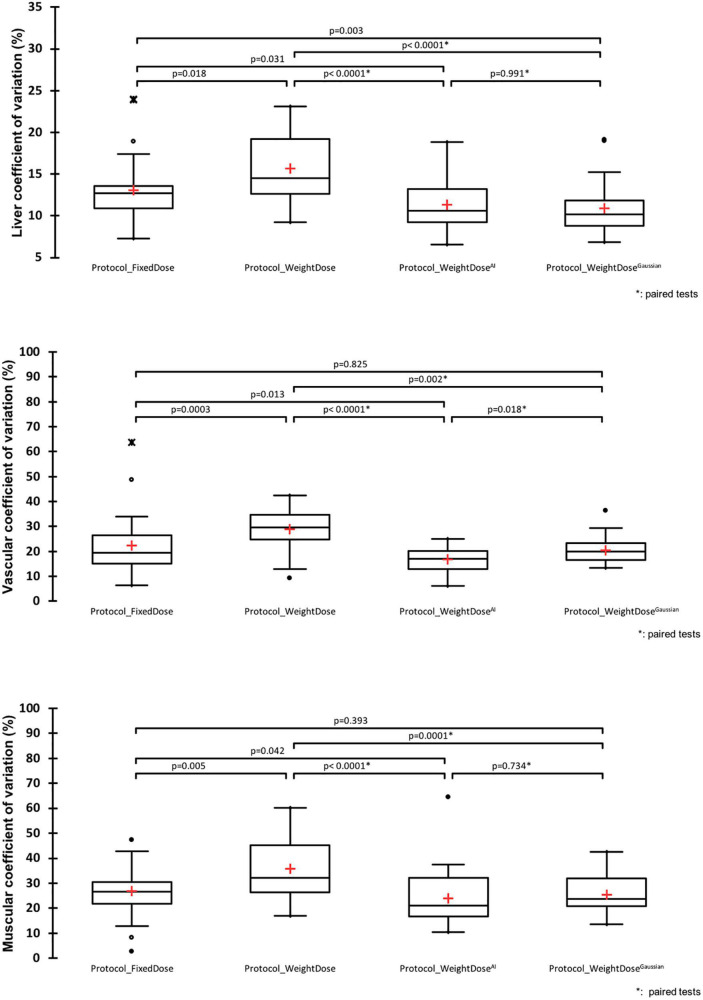
Image noise analysis. *Paired Wilcoxon tests were used to compare *protocol_WeightDose* and protocol_Weight dose^AI^ data: Otherwise Mann–Whitney tests were used.

Two patients in the *protocol_FixedDose* group had diffuse liver metastatic involvement that did not allow their hepatic CV to be calculated. Overall, *protocol_WeightDose* led to noisier images with higher liver, vascular and muscular CVs (Figure 1). The mean liver CVs were equal to 15.57% ± 4.32 vs. 13.04% ± 3.51 for *protocol_WeightDose* and *protocol_FixedDose*, respectively (*p* = 0.018). Mean vascular CVs were 28.67% ± 8.65 vs. 22.25% ± 10.37 for *protocol_WeightDose* and *Protocol_FixedDose*, respectively (*p* = 0.0003). Mean muscular CVs were 35.87% ± 12.46 vs. 26.86% ± 8.63 for *protocol_WeightDose* and *Protocol_FixedDose*, respectively (*p* = 0.0.005).

### Gaussian filter width determination for the *protocol_WeightDose*^Gaussian^

The GPF width to be applied to the *protocol_WeightDose* acquisitions was determined from the NEMA-NU2 phantom acquisitions to ensure equivalent noise as compared to the *protocol_FixedDose*. A 2.6 mm GPF width was highlighted by dichotomization, applied and used thereafter. NEMA-NU2 CVs were equal to 23.15, 27.63 and 23.30% for *protocol_FixedDose, protocol_WeightDose*, and *Protocol_WeightDose^Gaussian^*, respectively.

### Performances of *protocol_WeightDose*^AI^ and *protocol_WeightDose*^Gaussian^

#### Image quality: Noise and contrast

On paired comparison, *protocol_WeightDose^AI^* led to less noisy images than *protocol_WeightDose* with lower liver, vascular and muscular CVs (Figure 1). Mean liver, vascular and muscular CVs were 11.42% ± 3.05 vs. 15.57% ± 4.32 (*p* < 0.0001), 16.62% ± 6.40 vs. 28.67% ± 8.65 (*p* < 0.0001) and 23.88% ± 10.58 vs. 35.87% ± 12.46 (*p* < 0.0001), respectively. Moreover, mean liver, vascular and muscular CVs using *protocol_WeightDose^AI^* were slightly lower from those of *protocol_FixedDose* (Figure 1).

On paired comparison, *protocol_WeightDose^Gaussian^* also led to less noisy images than *protocol_WeightDose* with lower liver, vascular and muscular CVs (Figure 1). *Protocol_WeightDose^Gaussian^* mean liver, vascular and muscular CVs were 10.92% ± 3.00 (*p* < 0.0001), 20.50% ± 5.12 (*p* = 0.002) and 25.49% ± 7.14 (*p* = 0.0001), respectively. The mean liver CV obtained with the *protocol_WeightDose^Gaussian^* protocol was also lower than with the *protocol_FixedDose*. However, mean vascular and muscular CVs were not different (Figure 1). There were no significant differences between mean liver and muscular CVs of the *protocol_WeightDose^AI^ and* the *protocol_WeightDose^Gaussian^.* In contrast, the mean vascular CV of the *protocol_WeightDose^Gaussian^* was higher than that of the *protocol_WeightDose^AI^*, *p* = 0.018 (Figure 1).

On paired comparison, tumour-to-background ratios and tumour-to-liver ratios were lower when using *protocol_WeightDose^AI^* with a mean tumour-to-background ratio of 6.78 ± 3.49 vs. 7.57 ± 4.73 for the *protocol_WeightDose* (*p* = 0.04) and a mean tumour-to-liver ratio of 5.96 ± 5.43 vs. 6.77 ± 6.19 (*p* = 0.0001). Using the *protocol_WeightDose^Gaussian^* both these ratios were also lower than those obtained with the *protocol_WeightDose*, and even lower than those obtained with the *protocol_WeightDose^AI^*. The mean tumour-to-background ratio was equal to 5.60 ± 2.95 (*p* < 0.0001 as compared to *protocol_WeightDose* and *p* = 0.013 as compared to protocol_WeightDose^AI^) and the mean tumour-to-liver ratio was equal to 5.22 ± 4.93 (*p* < 0.0001 as compared to *protocol_WeightDose* and *p* = 0.02 as compared to protocol_WeightDose^AI^).

#### Lesions quantitative values

**FIGURE 2:**
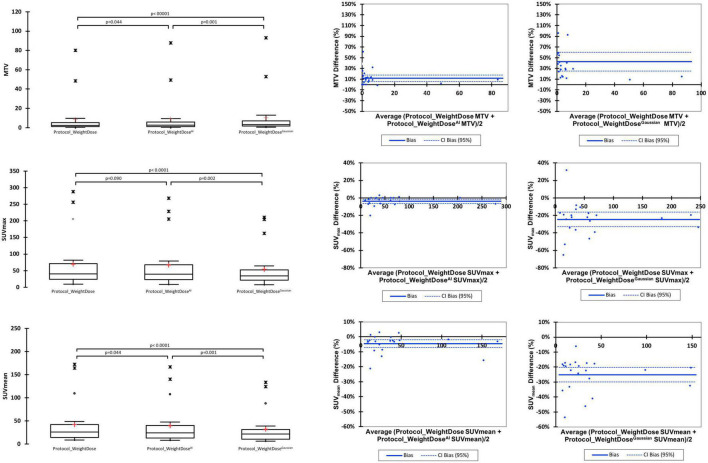
Paired comparison of *protocol_WeightDose, protocol_WeightDose^AI^*, and *protocol_WeightDose^Gaussian^* quantitative values.

Metabolic tumour volumes, SUV_max_ and SUV_mean_ of the hottest lesion were different between *protocol_WeightDose* and *protocol_WeightDose^AI^* on paired comparison. Similar findings were observed between *protocol_WeightDose* and *protocol_WeightDose^Gaussian^* (Figure 2).

Metabolic tumour volumes were significantly higher when using *protocol_WeightDose^AI^* with a mean MTV of 9.11 ± 20.26 vs. 8.46 ± 18.87 for the *protocol_WeightDose* (*p* = 0.044). *Protocol_WeightDose^Gaussian^* led to even higher MTV values (10.41 ± 21.44) with a *p*-value < 0.0001 as compared to *protocol_WeightDose* and equal to 0.001 as compared to *protocol_WeightDose^AI^*.

SUV_max_ and SUV_mean_ were lower for the *protocol_WeightDose ^AI^* with a mean SUV_max_ of 66.65 ± 71.97 vs. 69.76 ± 77.29 for the *protocol_WeightDose* (*p* = 0.09) and a mean SUV_mean_ equal to 39.67 ± 42.95 vs. 41.72 ± 46.42 for the *protocol_WeightDose* (*p* = 0.044) (Figure 2). *Protocol_WeightDose^Gaussian^* led to even lower SUV values than protocol *protocol_WeightDose ^AI^*: 54.06 ± 59.11 for SUV_max_ (*p* = 0.002) and 32.32 ± 35.76 for SUV_mean_ (*p* = 0.001).

The mean % differences in MTV, SUV_max_ and SUV_mean_ before and after denoising by application of the *protocol_WeightDose^AI^* were low, equal to +11.14% (95% CI = 4.84–17.43), −3.92% (95% CI = −6.25 to −1.59) and −4.32% (95% CI = −6.98 to −1.66), respectively (Figure 2). These mean % differences were higher by using the *Protocol_WeightDose^Gaussian^*: + 42.69% (95% CI = 25.23–60.15) for MTV, −24.66% (95% CI = −33.02 to −16.29) for SUV_max_ and −25.08 (95% CI = −30.00 to −20.15%) for SUV_mean_.

**FIGURE 3:**
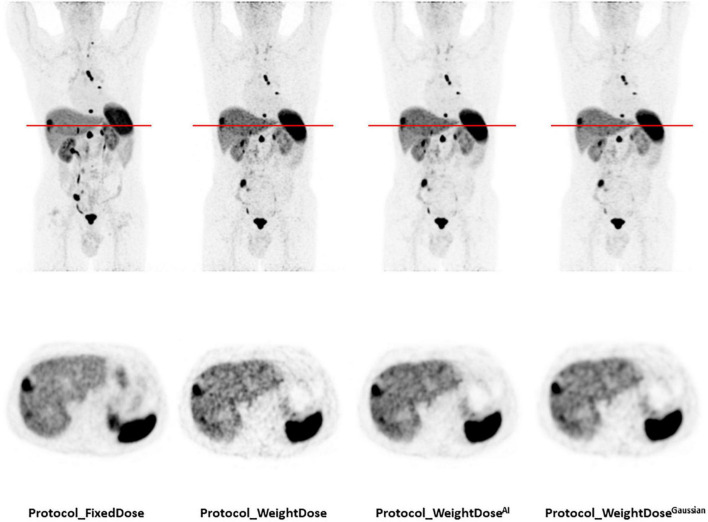
Representative images of a patient who underwent all four protocols during the inclusion period. A total of 77-year-old man of normal weight (BMI = 21.3 kg/m^2^) with a well-differentiated metastatic pancreatic neuroendocrine tumour (grade 1, Ki 67 < 1%). Injected doses were 158 MBq (2.4 MBq/kg) for *protocol_FixedDose* and 86 MBq (1.3 MBq/kg) for *protocol_WeightDose*, *protocol_WeightDose^AI^* and protocol_*WeightDose*^Gaussian^. All images are scaled to the same SUV_max_.

Side-by-side representative images of a patient who underwent all four protocols during the inclusion period are displayed in Figure 3. Complete data for the nine patients who had all protocols are reported in Supplementary Table 1.

## Discussion

This study shows that the degradation of PET image quality due to a reduction in injected dose at the end of the ^68^Ge/^68^Ga generator lifetime can be counterbalanced effectively by using AI-based PET denoising.

The EANM guidelines recommend an administered activity ranging from 100 to 200 Mbq, meaning that both fixed dose and ponderal dose strategies can be considered (16). To date, these two strategies have not been compared and the use of either one is at the discretion of each nuclear medicine department.

In our center, at the start of the generator lifetime using the *protocol_FixedDose*, almost all patients were injected with more than 1.5 MBq/kg of ^68^Ga-DOTATOC. This explains the better image quality parameters observed with *Protocol_FixedDose* than with *Protocol_WeightDose.* The use of *Protocol_WeightDose^AI^* or *Protocol_WeightDose^Gaussian^* led to an increase in image quality comparable to that of our former *protocol_FixedDose* with regard to image noise. To achieve comparable noise image quality performances at the end of the generator lifetime as per *Protocol_FixedDose* taken as reference in the present study, there are four possible solutions: (i) increasing the injected dose to 2.0 MBq/kg, which corresponds to the mean injected dose when using *Protocol_FixedDose*; (ii) increasing the PET acquisition time to compensate for the lower injected dose; (iii) adapting the reconstruction parameters, i.e., applying a Gaussian Filter; or (iv) exploring external solutions such as AI-based post-reconstruction PET denoising software.

Increasing the injected dose does not seem feasible as the eluted dose will inevitably decrease over time. Furthermore, it is always preferable for the patient’s sake to decrease rather than increase the injected dose (8, 9). Increasing the acquisition time seems illusory in busy PET units, especially considering the short and therefore restrictive half-life of ^68^Ga. The use of a Gaussian filter during reconstruction can certainly solve the problem of image noise but is detrimental to the quantitative values of the lesions. In the present study, the tumour volumes are overestimated on average by more than 40% and the SUVs underestimated by more than 20%, which does not seem tolerable in clinical settings. This is consistent with previous results obtained with FDG-PET (12). Thus, applying PET denoising software to a *Protocol_WeightDose* would provide good noise quality and quantitatively less altered ^68^Ga-DOTATOC PET/CT images acquired rapidly and at “low-dose.” From an economic point of view, the costs of using an AI-based PET denoising solution should offset the costs related to the decreasing yield of the generator. As more and more ^68^Ga-labeled tracers will probably be commercialized in the future, the value of AI will increase.

Previous work from our group on AI-based PET denoising in a large series of FDG PET scans showed the reassuringly high concordance rate in lesion detection between conventional and AI-processed PET images in the same patient (11). Therefore, the primary aim of PET imaging, which is lesion detection with high sensitivity, does not seem to be jeopardized by AI. Although FDG- and ^68^Ga-labeled tracers target different diseases and show differences in biodistribution, we feel it is safe to extrapolate the detection rate obtained in AI-processed FDG PET scans to AI-processed ^68^Ga PET scans, as the tumour contrast in the latter is often much higher than in the former. Also, the article by Liu et al. focusing on a cross-tracer and cross-protocol deep transfer learning method for noise reduction indicated that the network trained with FDG datasets can effectively reduce noise in low-dose PET images from less commonly used tracers (i.e., ^68^Ga-DOTATATE) while preserving diagnostic information (18).

We used two methods to evaluate tumour contrast: The tumour-to-background ratio using a doughnut-shaped VOI and the tumour-to-liver ratio. For the doughnut-shaped VOI, the choice of the tumour-contouring method was crucial to ensure the reliability of the resulting background noise measurements. We chose to use a thresholding value set in reference to SUV_max_, which was previously demonstrated in the study by Reddy et al. (19) to be the most accurate measurement when compared to morphological volumes. Beyond tumour detectability, one must also take into account the risk of false positive results which increases with the noise in the image. In particular, an increase in liver background noise can easily lead to the overestimation of hepatic metastatic involvement by taking noise for small lesions, especially in patients followed for neuroendocrine tumours with high hepatic metastatic risk. Figure 3 illustrates this issue nicely.

We acknowledge our study has limitations. First, the use of semi-quantitative parameters for ^68^Ga-peptide imaging has some limitations, although it is the most commonly used method in practice (20, 21). One of the main limitations is that it is subject to variations in PET device sensitivity, image acquisition parameters and patient-specific factors that can lead to inaccuracies in quantification (22). Another limitation is that it relies on the assumption that the tracer uptake is proportional to the density of the target receptor, which may not always be the case (23, 24). Secondly, this is a single-center study on ^68^Ga-DOTATOC PET images only. Although the cohort was small, it covers the lifetime of one generator, i.e., a period of approximately 1 year, during which all patients were included. The robustness of our findings need to be investigated in a multicenter study on different PET systems. Thirdly, only the *protocol_WeightDose* PET scans were AI-processed, leading to a limited number of pairwise comparisons. However, at the start of the generator lifetime, we did not feel the need to use AI processing in view of the good image quality of the *protocol_FixedDose* PET scans. The need to improve image quality became evident at the end of generator life. Finally, we could not properly evaluate the SUV_peak_ data because the small target lesions occurring in 57.7% of *protocol_FixedDose* patients (15/26) and 81.8% of *protocol_WeightDose* and *protocol_WeightDose^AI^* patients (18/22) (25) were not sufficiently measurable. This was because most target lesions were small with a mean MTV around only 9cc for *protocol_WeightDose* and *protocol_WeightDose^AI^*.

## Conclusion

The degradation of PET image quality due to a reduction of injected dose at the end of the ^68^Ge/^68^Ga generator lifespan can be counterbalanced effectively by using an AI-based PET denoising solution.

## Data availability statement

The datasets generated during and/or analyzed during the current study are available from the corresponding author on reasonable request.

## Ethics statement

The studies involving human participants were reviewed and approved by the Ethics Committee of Centre François Baclesse. The patients/participants provided their written informed consent to participate in this study.

## Author contributions

EQ and CL performed the image reading and wrote the first draft of the manuscript. All authors commented on previous versions of the manuscript, read, and approved the final manuscript, contributed to the study’s conception and design, material preparation, data collection, and analysis were performed.

## Supplementary material

Supplementary Figure 1 Injected dose scatter plot according to BMI for the 44 patients in *Protocol_FixedDose* group.
