# Supplementary material for: Artificial intelligence-based 68Ga-DOTATOC PET denoising for optimizing 68Ge/68Ga generator use throughout its lifetime
Source: Front Med (Lausanne). 2023 Mar 13;10:1137514. doi: 10.3389/fmed.2023.1137514 (PMC10040856; doi:10.3389/fmed.2023.1137514)
Supplement: Supplementary file 1 [file Table_1.DOCX]

**Supplemental Table 1:** Clinical and quantitative data of the 9 patients who had all protocols during the inclusion period.

| **N°** | **Sex** | **Age (year)** | **BMI (kg/m²)** | **Primary tumour location** | **Protocol type** | **Delay between acquisitions (months)** | **Injected Dose**  **(Mbq/kg)** | **CoV liver (%)** | **CoV Aorta (%)** | **CoV muscle (%)** | **VOI location** | **MTV**  **(cc)** | **SUV_max_** | **SUV_mean_** | | **T/bckg** | | **T/liver** |
| --- | --- | --- | --- | --- | --- | --- | --- | --- | --- | --- | --- | --- | --- | --- | --- | --- | --- | --- |
| 37 | F | 67 | 24.0 | Small bowel | FixedDose | 7 | 2.5 | 9.0 | 6.3 | 24.0 | Liver | 3.95 | 64.23 | 38.48 | 5.64 | | 10.0 | |
|  |  |  |  |  | WeightDose |  | 1.6 | 14.9 | 9.4 | 30.1 |  | 3.27 | 62.69 | 37.23 | 5.51 | | 8.9 | |
|  |  |  |  |  | WeightDose^IA^ |  | 1.6 | 9.7 | 6.2 | 19.6 |  | 3.72 | 60.58 | 36.23 | 5.09 | | 8.1 | |
|  |  |  |  |  | WeightDose^Gaussian^ |  | 1.6 | 11.1 | 16.6 | 25.9 |  | 3.83 | 52.43 | 31.27 | 4.63 | | 7.4 | |
| 38 | F | 49 | 18.1 | Small bowel | FixedDose | 4 | 1.6 | 14.0 | 33.9 | 25.7 | Peritoneal | 0.26 | 7.97 | 7.37 | 2.21 | | 1.7 | |
|  |  |  |  |  | WeightDose |  | 1.6 | 12.7 | 24.9 | 30.7 |  | 0.03 | 9.09 | 8.9 | 2.06 | | 1.6 | |
|  |  |  |  |  | WeightDose^IA^ |  | 1.6 | 9.3 | 11.8 | 17.9 |  | 0.30 | 8.82 | 8.64 | 1.91 | | 1.5 | |
|  |  |  |  |  | WeightDose^Gaussian^ |  | 1.6 | 8.7 | 15.5 | 21.7 |  | 0.75 | 7.65 | 6.20 | 1.73 | | 1.1 | |
| 41 | M | 77 | 21.6 | Pancreas | FixedDose | 7 | 2.4 | 16.6 | 11.5 | 21.8 | Node | 1.28 | 55.36 | 32.92 | 8.62 | | 8.1 | |
|  |  |  |  |  | WeightDose |  | 1.3 | 19.0 | 13.7 | 41.8 |  | 1.34 | 41.69 | 23.62 | 8.14 | | 4.8 | |
|  |  |  |  |  | WeightDose^IA^ |  | 1.3 | 14.6 | 12.1 | 24.7 |  | 1.65 | 39.06 | 22.4 | 8.06 | | 4.3 | |
|  |  |  |  |  | WeightDose^Gaussian^ |  | 1.3 | 8.8 | 13.4 | 23.6 |  | 2.04 | 28.80 | 19.96 | 7.59 | | 3.9 | |
| 42 | M | 33 | 23.2 | Stomach | FixedDose | 7 | 2.3 | 19.6 | 17.5 | 19.6 | No lesion |  |  |  |  | |  | |
|  |  |  |  |  | WeightDose |  | 1.5 | 25.6 | 42.4 | 25.6 |  |  |  |  |  | |  | |
|  |  |  |  |  | WeightDose^IA^ |  | 1.5 | 16.4 | 20.3 | 16.4 |  |  |  |  |  | |  | |
|  |  |  |  |  | WeightDose^Gaussian^ |  | 1.5 | 8.7 | 19.9 | 20.8 |  |  |  |  |  | |  | |
| 48 | F | 82 | 22.6 | Breast | FixedDose | 4 | 2.9 | 29.1 | 17.4 | 29.1 | No lesion |  |  |  |  | |  | |
|  |  |  |  |  | WeightDose |  | 1.6 | 36.2 | 25.8 | 36.2 |  |  |  |  |  | |  | |
|  |  |  |  |  | WeightDose^IA^ |  | 1.6 | 20.9 | 15.5 | 20.9 |  |  |  |  |  | |  | |
|  |  |  |  |  | WeightDose^Gaussian^ |  | 1.6 | 13.2 | 13.7 | 25.0 |  |  |  |  |  | |  | |
| 49 | M | 80 | 29.1 | Small bowel | FixedDose | 6 | 2.0 | 23.9 | 30.5 | 47.4 | Small bowel | 1.58 | 22.86 | 13.41 | 6.24 | | 3.3 | |
|  |  |  |  |  | WeightDose |  | 2.0 | 22.3 | 34.7 | 49.2 |  | 2.30 | 16.42 | 9.62 | 4.83 | | 2.1 | |
|  |  |  |  |  | WeightDose^IA^ |  | 2.0 | 16.6 | 20.9 | 33.7 |  | 2.61 | 16.13 | 9.30 | 4.97 | | 1.9 | |
|  |  |  |  |  | WeightDose^Gaussian^ |  | 2.0 | 19.0 | 24.8 | 35.5 |  | 3.05 | 13.51 | 7.94 | 4.27 | | 1.6 | |
| 50 | M | 68 | 30.8 | Small bowel | FixedDose | 6 | 1.7 | 12.9 | 19.2 | 22.3 | Liver | 3.13 | 27.62 | 16.42 | 2.93 | | 3.7 | |
|  |  |  |  |  | WeightDose |  | 1.5 | 20.2 | 24.2 | 48.7 |  | 2.26 | 31.95 | 18.56 | 3.50 | | 4.7 | |
|  |  |  |  |  | WeightDose^IA^ |  | 1.5 | 12.7 | 12.9 | 26.6 |  | 2.49 | 31.95 | 17.99 | 3.46 | | 4.2 | |
|  |  |  |  |  | WeightDose^Gaussian^ |  | 1.5 | 12.6 | 15.6 | 33.3 |  | 3.22 | 25.56 | 14.84 | 2.96 | | 3.5 | |
| 55 | M | 58 | 25.2 | Small bowel | FixedDose | 6 | 1.9 | 25.7 | 26.8 | 25.7 | No lesion |  |  |  |  | |  | |
|  |  |  |  |  | WeightDose |  | 1.9 | 49.2 | 26.7 | 49.2 |  |  |  |  |  | |  | |
|  |  |  |  |  | WeightDose^IA^ |  | 1.9 | 32.2 | 16.1 | 32.2 |  |  |  |  |  | |  | |
|  |  |  |  |  | WeightDose^Gaussian^ |  | 1.9 | 10.4 | 23.8 | 25.7 |  |  |  |  |  | |  | |
| 63 | F | 70 | 31.8 | Small bowel | FixedDose | 6 | 1.7 | 10.8 | 15.3 | 16.5 | Bone | 0.24 | 22.96 | 13.97 | 11.64 | | 3.3 | |
|  |  |  |  |  | WeightDose |  | 1.7 | 18.8 | 22.3 | 43.9 |  | 0.30 | 21.55 | 12.98 | 13.01 | | 2.0 | |
|  |  |  |  |  | WeightDose^IA^ |  | 1.7 | 12.0 | 10.6 | 23.9 |  | 0.39 | 17.60 | 10.49 | 10.59 | | 1.5 | |
|  |  |  |  |  | WeightDose^Gaussian^ |  | 1.7 | 11.5 | 17.2 | 17.2 |  | 0.54 | 12.48 | 7.49 | 1.00 | | 1.1 | |
