# Supplementary figures and images for: Artificial intelligence-based 68Ga-DOTATOC PET denoising for optimizing 68Ge/68Ga generator use throughout its lifetime
Source: Front Med (Lausanne). 2023 Mar 13;10:1137514. doi: 10.3389/fmed.2023.1137514 (PMC10040856; doi:10.3389/fmed.2023.1137514)

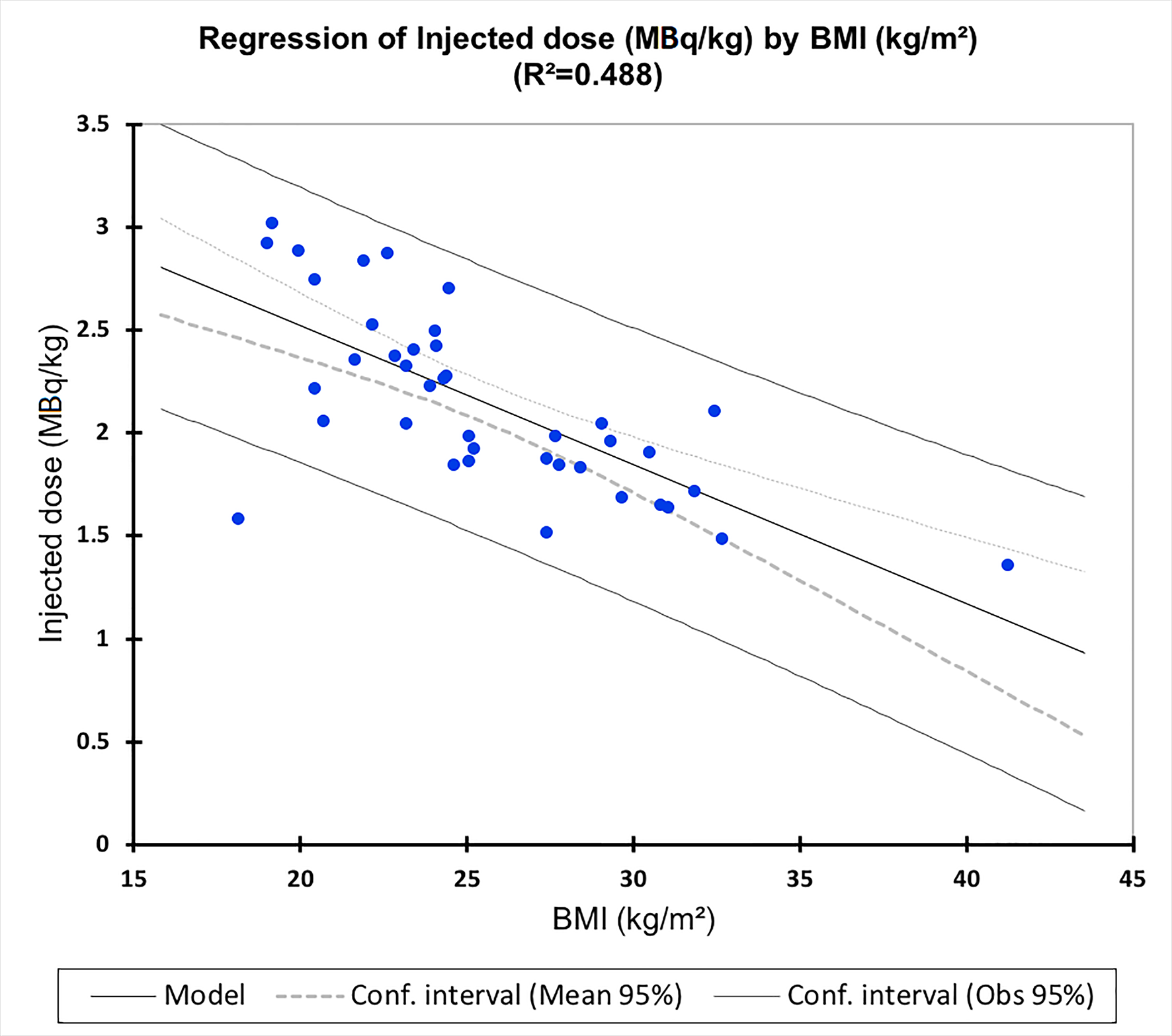

Supplement: Supplementary Figure 1 — Injected dose scatter plot according to BMI for the 44 patients in Protocol_FixedDose group. [file Image_1.TIF]
